# Identifying differentially expressed proteins in sorghum cell cultures exposed to osmotic stress

**DOI:** 10.1038/s41598-018-27003-1

**Published:** 2018-06-06

**Authors:** Rudo Ngara, Elelwani Ramulifho, Mahsa Movahedi, Nemera G. Shargie, Adrian P. Brown, Stephen Chivasa

**Affiliations:** 1 Department of Plant Sciences, University of the Free State, Qwaqwa Campus, P. Bag X13, Phuthaditjhaba, South Africa; 2 Agricultural Research Council-Small Grain Institute, P. Bag X29, Bethlehem, 9700 South Africa; 3 Department of Biosciences, Durham University, South Road, Durham, DH1 3LE United Kingdom; 4 Agricultural Research Council-Grain Crops Institute, P. Bag X1251, Potchefstroom, 2520 South Africa

## Abstract

Drought stress triggers remarkable physiological changes and growth impediments, which significantly diminish plant biomass and crop yield. However, certain plant species show notable resilience, maintaining nearly normal yields under severe water deficits. For example, sorghum is a naturally drought-tolerant crop, which is ideal for studying plant adaptive responses to drought. Here we used sorbitol treatments to simulate drought-induced osmotic stress in sorghum cell suspension cultures and analysed fractions enriched for extracellular matrix proteins using isobaric tags for relative and absolute quantification technology. Sorbitol induced an overall increase in protein secretion, with putative redox proteins, proteases, and glycosyl hydrolases featuring prominently among the responsive proteins. Gene expression analysis of selected candidates revealed regulation at the transcriptional level. There was a notable differential gene expression between drought-tolerant and drought-sensitive sorghum varieties for some of the candidates. This study shows that protein secretion is a major component of the sorghum response to osmotic stress. Additionally, our data provide candidate genes, which may have putative functions in sorghum drought tolerance, and offer a pool of genes that could be developed as potential biomarkers for rapid identification of drought tolerant lines in plant breeding programs.

## Introduction

Water is an essential solvent for cell biochemical reactions and is indispensable for life. Extreme dehydration reduces cell turgor and adversely affects cellular metabolic processes. Prolonged water deficits, such as imposed by severe droughts, result in leaf wilting and ultimately ends in plant death. While the majority of plants are very sensitive to water loss and capitulate under drought stress, several plant species have genetic adaptations ensuring their survival in marginal lands and extreme environments with limited water. There is intense research interest in understanding the molecular responses of plants to drought stress.

Upon sensing soil water deficits, plants activate transcriptional changes enabling them to deploy mechanisms for conserving water, metabolic reprogramming for adaptation to drought stress, and redirection of growth patterns to follow moisture gradients. The signalling events underpinning the adaptive responses to drought are complex and involve abscisic acid (ABA)-dependent and ABA-independent pathways. Dehydration triggers the biosynthesis of ABA^1^, which regulates plant water balance and osmotic stress tolerance via control of stomatal aperture^2^ and activation of stress tolerance genes^3^. ABA binds to its soluble receptor complex, pyrabactin resistance1/PYR1-Like/regulatory component of PYR1/PYRL/RCAR ABA receptors^4,5^. Receptor binding inhibits protein phosphatase 2C activity^4–11^, triggering autophosphorylation of SnRK2 kinases^12–14^, which in turn phosphorylate numerous substrates and activate multiple pathways including guard cell closure and drought stress-adaptive gene expression^15^.

A conserved ABA-responsive element in the gene promoter is an essential *cis*-acting element for regulating ABA-inducible gene expression^16^. MYB and MYC recognition sites are additional *cis*-acting elements identified in the promoters of some ABA-regulated genes^17^. Activation of ABA-dependent pathways in transgenic Arabidopsis by constitutive overexpression of the transcription factors ABF2, MYC2, or MYB2, leads to improved tolerance to drought/osmotic stress^18,19^. ABA-independent signalling pathways also operate in activation of stress-responsive genes during drought. Neither the primary receptors involved nor the signalling components that lead to drought-induced gene expression via ABA-independent pathways are known. However, the responsive genes possess a conserved *cis*-acting element in the promoter sequence known as the dehydration-responsive element (DRE)^20^. DRE-binding Protein 2A (DREB2A) specifically binds the DRE sequence^21^ to activate Arabidopsis gene expression in response to drought, high salinity, and heat-shock stress^21,22^. Constitutive activation of the ABA-independent pathways by overexpression of DREB2A confers increased drought tolerance in Arabidopsis^21^.

Transcriptomic changes driven by drought-induced signalling reprogram the proteome and cellular metabolism. The functional significance of most of the proteins is not fully understood. However, some of these have a role in signal transduction and activation of further gene expression, while others clearly support the adaptive response strategy to re-establish cellular homeostasis and survival under drought stress. The classes of proteins deployed during plant adaptation to drought were reviewed by Shinozaki and Yamaguchi-Shinozaki^3^. They include aquaporins for water movement across membranes and enzymes for the biosynthesis of osmolyte sugars, proline, and glycine-betaine, which are important for osmotic rebalancing. Cellular detoxification enzymes, such as ascorbate peroxidase, glutathione-S-transferase, catalase, and superoxide dismutase prevent oxidative damage, while protection of membranes and macromolecules is maintained by chaperones, messenger RNA-binding proteins, late embryogenesis abundant proteins, and similar proteins. The adaptive reprogramming of the transcriptome and proteome is supported by increased protein turnover facilitated by enzymes and proteins, such as ubiquitin, Clp protease, and thiol proteases. Transgenic plants overexpressing some of these genes acquire drought tolerance^23^, indicating that the gene products really function in stress tolerance.

Most of the research into plant molecular responses to drought has been conducted using drought-sensitive model species, such as *Arabidopsis thaliana*. Sorghum (*Sorghum bicolor* L. Moench), a naturally drought tolerant cereal^24^ with high genetic diversity, is a good model system for studying drought stress-adaptive responses^25^, especially with a view to identify novel genes that could be used to generate drought tolerant crops. The sorghum genome has been sequenced^26^ and some transcriptomic^27^ and proteomic^28^ analysis of leaf responses to osmotic stress and drought have been reported. We have a longstanding interest in understanding how the extracellular matrix proteome changes during stress-adaptive responses^29,30^. Our hypothesis is that the extracellular matrix is a repository of signal molecules used for cell-cell communications during stress adaptation, and analysis of this compartment may lead to identification of signal-regulatory proteins with a pivotal role in drought tolerance. Here, we used a sorghum cell suspension culture system to identify differentially expressed proteins in the extracellular matrix during osmotic stress and show that selected targets are differentially expressed in drought-tolerant and sensitive sorghum lines during drought stress.

## Results

### Identification of sorghum cell suspension culture ECM proteins

**Figure 1:**
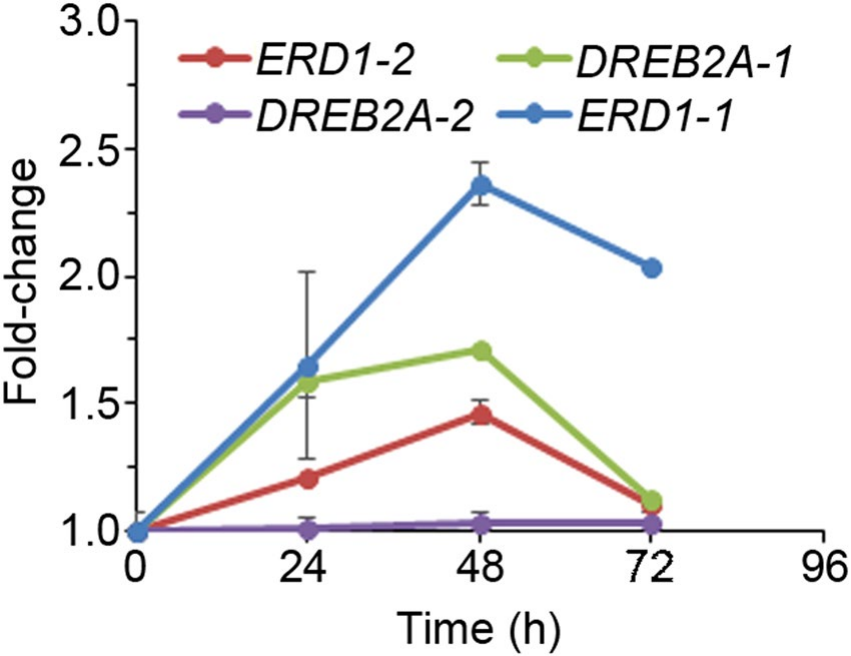
Activation of sorghum *ERD1* and *DREB2A* expression in response to sorbitol. Sorghum cell suspension cultures were treated with sorbitol and cells harvested at the indicated time-points. Gene expression was analysed using qRT-PCR. Error bars represent means ± S.D. (*n* = 3).

We designed experiments to isolate fractions enriched for secreted proteins in the soluble phase of the sorghum extracellular matrix (ECM). Our goal was to identify these proteins and analyse their response to osmotic stress. We used sorghum cell suspension cultures as a source of easily extractable soluble ECM proteins from the culture growth medium. Basing on preliminary data obtained from the growth curve, we used exponential phase 8-day-old cultures for stress treatments. Sorghum cell cultures were treated with 400 mM sorbitol^31^ and cells harvested every 24 h until 72 h for RNA extraction. We analysed expression profiles of sorghum homologues of Arabidopsis drought marker genes, *ERD1* and *DREB2A*, to monitor the osmotic stress response and establish the optimal time for harvesting cells for protein extraction. We identified sorghum homologues of Arabidopsis ERD1 and DREB2A, which we named ERD1-1 (SORBI_3004G162400), ERD1-2 (SORBI_3006G065100), DREB2A-1 (SORBI_3009G101400), and DREB2A-2 (SORBI_3003G058200). With the exception of *DREB2A-2*, all the genes were activated by sorbitol treatment, with expression peaking at 48 h (Fig. 1). Therefore, in subsequent experiments, 48 h was selected as the time after sorbitol addition to harvest cell cultures for protein extraction. Use of 4 biological replicates for both sorbitol treatments and controls ensured that proteins with highly reproducible responses were identified.

Cell cultures were treated with sorbitol and secreted proteins were isolated from the culture medium by simple filtration of the cell culture and acetone precipitation of the filtrate. ECM protein samples from control and osmotic stressed cultures were then digested with trypsin, labelled with iTRAQ, fractionated by liquid chromatography, and analysed using tandem mass spectrometry. Only proteins with at least 2 sequenced peptides, each with a statistical confidence threshold ≥ 95%, were considered positively identified. A total of 179 different proteins were positively identified in the ECM fractions of sorghum cell cultures. The full mass spectrometry data of these proteins is provided in Supplementary Dataset (Table S2). This dataset represents a snapshot of the sorghum cell culture secretome at 10 days post-subculturing. Some of the 179 proteins have functional annotations in the protein database derived from sequence identity, which include peroxidases, alpha-galactosidases, alpha-mannosidase, endoglucanases, purple acid phosphatase, malate dehydrogenase and xyloglucan endotransglucosylase. Other proteins are annotated as uncharacterized proteins since database annotation is still incomplete. All the functionally annotated and uncharacterized proteins identified here will require experimental validation of protein function. Apart from the sorghum specific proteins, we also identified trypsin and human keratin proteins, which are known contaminants in proteomic analysis. These contaminants serve as defacto positive controls and their identification in interrogating extensive protein databases indicates that protein identification was specific.

### Differentially expressed ECM proteins in response to osmotic stress

For quantitative analysis of osmotic stress-related protein expression, a minimum threshold of 2-fold change in protein abundance at a significance level of *p* ≤ 0.05 was applied to filter the dataset. This resulted in a total of 92 proteins that were differentially expressed in response to sorbitol-induced osmotic stress (Table 1). With the exception of one down-regulated protein, the rest were up-regulated, indicating that sorbitol triggered an overall increase in protein secretion. Next we used the SignalP tool to analyse the protein sequences for the presence of a signal peptide, which targets proteins to the secretory pathway. A predicted N-terminal signal peptide was identified in 54 of these proteins (Table 1), indicating that they are secreted via the classical secretory pathway requiring a leader sequence. The remaining proteins were predicted not to have an N-terminal signal peptide (Table 1). Bioinformatic analysis of the primary sequences was used to detect putative functional domains in the differentially expressed proteins, which were then assigned to specific protein families (Table 1). There were 18 proteins assigned to glycosyl-hydrolases/glycosidases, 5 to cell wall modifying enzymes, 12 to proteases, 27 to redox proteins, and 30 proteins were left unclassified.

**Table 1:** List of sorghum secreted proteins that are responsive to sorbitol-induced osmotic stress.

Prot. #^a^	Accession^b^	Protein Name	Ratio^c^	SD^d^	p value^e^	Signal Peptide^f^	Family name^g^
**Glycosyl-hydrolases/Glycosidases**							
6	A0A1B6QHZ6	Uncharacterized protein OS = *Sorghum bicolor* GN = SORBI_001G089000	2.93	0.12	4.85E-06	−	Glycoside hydrolase superfamily
8	C5X532	Alpha-galactosidase OS = *Sorghum bicolor* GN = SORBI_002G123100	2.05	0.05	7.96E-04	+	Glycoside hydrolase superfamily
22	A0A1B6QI05	Uncharacterized protein OS = *Sorghum bicolor* GN = SORBI_001G089100	2.19	0.08	1.79E-04	+	Glycoside hydrolase superfamily
27	C5XKE9	Endoglucanase OS = *Sorghum bicolor* GN = SORBI_003G015700	2.84	0.35	2.25E-05	−	Glycoside hydrolase family 9
28	C5Y397	Alpha-mannosidase OS = *Sorghum bicolor* GN = SORBI_005G132400	4.24	0.28	1.10E-06	+	Glycosyl hydrolase family 38
29	C5X8J4	Xyloglucan endotransglucosylase/hydrolase OS = *Sorghum bicolor* GN = SORBI_002G302000	3.58	0.24	1.42E-06	+	Xyloglucan endotransglucosylase/hydrolase
36	C5XB38	Uncharacterized protein OS = *Sorghum bicolor* GN = SORBI_002G055600	2.33	0.16	7.22E-05	+	Glycoside hydrolase family 18
72	C5X022	Uncharacterized protein OS = *Sorghum bicolor* GN = SORBI_001G525000	2.00	0.11	1.51E-04	+	Glycoside hydrolase family 28
82	A0A1B6QC86	Uncharacterized protein OS = *Sorghum bicolor* GN = SORBI_002G189100	2.07	0.16	6.77E-04	−	Glycoside hydrolase family 81
84	C5XFX7	Uncharacterized protein OS = *Sorghum bicolor* GN = SORBI_003G247000	2.29	0.20	5.08E-05	+	Glycoside hydrolase family 5
86	A0A1B6PTQ9	Uncharacterized protein OS = *Sorghum bicolor* GN = SORBI_005G204700	2.07	0.48	5.04E-03	+	Glycoside hydrolase family 28
88	C5XB39	Uncharacterized protein OS = *Sorghum bicolor* GN = SORBI_002G055700	2.35	0.11	1.15E-05	+	Glycoside hydrolase family 18
95	C5X5L7	Alpha-galactosidase OS = *Sorghum bicolor* GN = SORBI_002G417800	4.06	0.21	1.34E-06	+	Glycoside hydrolase family 27
133	C5WP48	Alpha-mannosidase OS = *Sorghum bicolor* GN = SORBI_001G268700	3.05	0.31	3.92E-04	+	Glycoside hydrolase family 38
140	C5YCY4	Uncharacterized protein OS = *Sorghum bicolor* GN = SORBI_006G160700	2.42	0.42	2.17E-04	−	Glycosyl hydrolase family 32
141	A0A1B6Q8G8	Uncharacterized protein (Fragment) OS = *Sorghum bicolor* GN = SORBI_003G440900	2.92	0.11	4.18E-05	−	Glycosyl hydrolase family 32
145	C5YBF1	Uncharacterized protein OS = *Sorghum bicolor* GN = SORBI_006G132700	2.79	0.29	2.59E-05	+	Glycoside hydrolase family 19
150	C5X3W3	Uncharacterized protein OS = *Sorghum bicolor* GN = SORBI_002G246400	2.30	0.42	1.16E-03	+	Glycoside hydrolase, family 28
**Cell wall modifying enzymes**							
2	C5WSF9	Uncharacterized protein OS = *Sorghum bicolor* GN = SORBI_001G301500	3.18	0.19	3.58E-06	+	Expansin/Lol pI
17	C5WSF0	Uncharacterized protein OS = *Sorghum bicolor* GN = SORBI_001G300800	3.23	0.41	3.53E-05	+	Expansin/Lol pI family
33	C5Z0P5	Uncharacterized protein OS = *Sorghum bicolor* GN = SORBI_009G055900	2.95	0,23	3.77E-06	−	Fasciclin-like arabinogalactan protein
59	C5WSE5	Uncharacterized protein OS = *Sorghum bicolor* GN = SORBI_001G300400	3.14	0.23	7.20E-06	+	Expansin/Lol pI
87	C5YVJ7	Uncharacterized protein OS = *Sorghum bicolor* GN = SORBI_009G232100	2.36	0.11	1.38E-06	+	Fasciclin 1 domain
**Proteases**							
14	A0A1B6PLA9	Uncharacterized protein OS = *Sorghum bicolor* GN = SORBI_006G104300	2.25	0.09	2.28E-05	+	Gamma-glutamyl-transpeptidase
20	A0A1B6QMT3	Uncharacterized protein OS = *Sorghum bicolor* GN = SORBI_001G348900	3.09	0.24	6.02E-06	+	Peptidase S10, serine carboxypeptidase
26	C5XQ74	Uncharacterized protein OS = *Sorghum bicolor* GN = SORBI_003G208800	2.05	0.10	4.70E-04	−	Aspartic peptidase A1 family
48	A0A1B6PNM7	Uncharacterized protein OS = *Sorghum bicolor* GN = SORBI_006G242000	3.27	0.13	7.96E-07	+	Peptidase C1A
85	C5WT64	Uncharacterized protein OS = *Sorghum bicolor* GN = SORBI_001G170700	2.05	0.18	4.15E-04	+	Peptidase S8 subtilisin-related
94	C5WXN2	Carboxypeptidase OS = *Sorghum bicolor* GN = SORBI_001G348800	2.10	0.14	2.50E-04	+	Peptidase S10, serine carboxypeptidase
98	C5YNA1	Uncharacterized protein OS = *Sorghum bicolor* GN = SORBI_007G172100	3.73	0.25	5.00E-06	+	Peptidase C1A
122	A0A1B6PHE0	Uncharacterized protein OS = *Sorghum bicolor* GN = SORBI_007G120800	3.13	0.41	8.07E-05	−	Peptidase M1 family
136	C5WQK1	Uncharacterized protein OS = *Sorghum bicolor* GN = SORBI_001G280000	2.77	0.28	3.61E-05	+	Peptidase S10, serine carboxypeptidase
138	A0A1B6QEG2	Uncharacterized protein OS = *Sorghum bicolor* GN = SORBI_002G315800	4.42	0.25	3.79E-07	+	Peptidase C1A
173	C5XDR4	Uncharacterized protein OS = *Sorghum bicolor* GN = SORBI_002G217200	2.87	0.25	8.52E-06	+	Peptidase C1A
178	C5Y171	Uncharacterized protein OS = *Sorghum bicolor* GN = SORBI_004G142800	5.62	0.52	1.25E-06	+	Peptidase C1A domain and family
**Redox proteins**							
7	A0A1B6QG95	Uncharacterized protein OS = *Sorghum bicolor* GN = SORBI_002G416600	2.08	0.03	6.75E-05	−	Plant peroxidase
13	C5Y360	Peroxidase OS = *Sorghum bicolor* GN = SORBI_005G011300	2.73	0.31	4.80E-05	+	Plant peroxidase
23	C5Z240	Uncharacterized protein OS = *Sorghum bicolor* GN = SORBI_010G003100	2.40	0.19	3.69E-05	+	Cupredoxin
30	C5WNY4	Uncharacterized protein OS = *Sorghum bicolor* GN = SORBI_001G129700	2.07	0.18	1.57E-04	+	Germin
31	C5YC92	Uncharacterized protein OS = *Sorghum bicolor* GN = SORBI_006G018100	2.18	0.30	3.17E-04	+	Germin
35	C5XIY1	Peroxidase OS = *Sorghum bicolor* GN = SORBI_003G152100	2.98	0.14	5.62E-06	+	Plant peroxidase
38	A0A1B6QN00	Uncharacterized protein OS = *Sorghum bicolor* GN = SORBI_001G360500	2.16	0.41	1.46E-03	+	Plant peroxidase
41	C6JSB7	Peroxidase OS = *Sorghum bicolor GN* = Sb0246s002010	7.79	1.84	4.99E-05	+	Plant peroxidase
51	A0A1B6QGB6	Uncharacterized protein OS = *Sorghum bicolor* GN = SORBI_002G416800	2.21	0.07	1.86E-05	+	Plant peroxidase
69	A0A1B6Q9F4	Uncharacterized protein OS = *Sorghum bicolor* GN = SORBI_002G057900	5.63	0.26	6.39E-08	−	Thioredoxin
92	C5XL59	Uncharacterized protein OS = *Sorghum bicolor* GN = SORBI_003G024700	−2.40	0.04	7.37E-04	−	Plant peroxidase
97	C5XIY0	Peroxidase OS = *Sorghum bicolor* GN = SORBI_003G152000	2.58	0.11	9.88E-06	−	Plant peroxidase
104	A0A194YU12	Uncharacterized protein OS = *Sorghum bicolor* GN = SORBI_004G341200	6.45	0.29	2.34E-08	−	Glutathione-disulphide reductase
110	A0A1B6QN96	Uncharacterized protein OS = *Sorghum bicolor* GN = SORBI_001G371900	13.59	1.99	1.14E-05	−	Cu-Zn superoxide dismutase-like
111	A0A1B6Q818	Uncharacterized protein OS = *Sorghum bicolor* GN = SORBI_003G416300	5.84	0.45	3.19E-07	−	GST C-terminal domain-like
129	C5X6P7	Uncharacterized protein OS = *Sorghum bicolor* GN = SORBI_002G140400	2.34	0.14	1.53E-05	+	Cupredoxin
131	C5WWQ2	Uncharacterized protein OS = *Sorghum bicolor* GN = SORBI_001G342600	8.47	2.43	6.43E-05	−	Thioredoxin
134	C5YQ75	Peroxidase OS = *Sorghum bicolor* GN = SORBI_008G010500	2.85	0.13	2.98E-06	+	Plant peroxidase
137	C5X780	Uncharacterized protein OS = *Sorghum bicolor* GN = SORBI_002G007200	2.70	0.12	9.70E-06	+	Cupredoxin
151	C5XC95	Uncharacterized protein OS = *Sorghum bicolor* GN = SORBI_002G345800	2.76	0.18	1.45E-05	+	Cupredoxin
155	A0A1B6QFT7	Uncharacterized protein OS = *Sorghum bicolor* GN = SORBI_002G392300	38.70	5.94	6.01E-06	+	Plant peroxidase
159	A0A1B6P9F6	Uncharacterized protein OS = *Sorghum bicolor* GN = SORBI_009G190800	4.28	0.35	1.95E-06	−	Thioredoxin
161	C5Z0N9	Peroxidase OS = *Sorghum bicolor* GN = SORBI_009G055300	2.76	0.11	9.30E-06	+	Plant peroxidase
167	C5XRU7	Uncharacterized protein OS = *Sorghum bicolor* GN = SORBI_004G148100	3.01	0.42	8.74E-05	+	Germin
169	A0A1B6QJR7	Uncharacterized protein OS = *Sorghum bicolor* GN = SORBI_001G189000	2.10	0.39	2.17E-03	−	Plant peroxidase
174	C5YN91	Uncharacterized protein OS = *Sorghum bicolor* GN = SORBI_007G171000	3.42	2.19	8.12E-03	−	FAD/NAD linked reductases, dimerization (C-terminal) domain
179	A0A1B6QB11	Uncharacterized protein OS = *Sorghum bicolor* GN = SORBI_002G133800	5.62	0.92	1.42E-05	−	FAD/NAD linked reductases, dimerization (C-terminal) domain
**Unclassified**							
19	A0A194YMM6	Uncharacterized protein OS = *Sorghum bicolor* GN = SORBI_010G262500	7.94	0.27	1.04E-08	−	Glyceraldehyde-3-phosphate dehydrogenase, type I
21	C5Z6U2	Uncharacterized protein OS = *Sorghum bicolor* GN = SORBI_010G210000	2.82	0.36	5.01E-05	−	Ubiquitin
40	C5XWE5	Uncharacterized protein OS = *Sorghum bicolor* GN = SORBI_004G197600	2.52	0.16	3.59E-05	+	Glycerophosphoryl diester phosphodiesterase family
43	A0A1B6PD28	Uncharacterized protein OS = *Sorghum bicolor* GN = SORBI_008G113000	3.08	0.18	7.65E-06	+	Purple acid phosphatase, N-terminal domain family
47	C5XPK9	Uncharacterized protein OS = *Sorghum bicolor* GN = SORBI_003G205600	2.69	0.09	8.53E-07	+	Leucine-rich repeat domain family
49	A0A194YGY2	Uncharacterized protein OS = *Sorghum bicolor* GN = SORBI_010G027000	5.90	0.35	1.21E-07	−	Enolase-like
53	C5Z6U1	Uncharacterized protein OS = *Sorghum bicolor* GN = SORBI_010G209900	2.96	0.16	5.80E-06	+	Not predicted
67	C5Y587	Uncharacterized protein OS = *Sorghum bicolor* GN = SORBI_005G049800	5.06	0.47	1.20E-06	−	Alginate lyase
68	C5YBH7	Uncharacterized protein OS = *Sorghum bicolor* GN = SORBI_006G135500	2.07	0.07	1.38E-04	+	Galactose oxidase central domain
70	C5XX52	Glyceraldehyde-3-phosphate dehydrogenase OS = *Sorghum bicolor* GN = SORBI_004G205100	4.49	0.29	5.19E-07	−	Glyceraldehyde 3-phosphate dehydrogenase
76	C5WXD7	Uncharacterized protein OS = *Sorghum bicolor* GN = SORBI_001G209300	2.67	0.13	2.62E-05	+	Uncharacterised protein family, basic secretory protein
79	C5X502	Dirigent protein OS = Sorghum bicolor GN = SORBI_002G119900	3.01	0.31	2.02E-05	+	Allene oxide cyclase/Dirigent protein
90	A0A1B6QEI0	Uncharacterized protein OS = *Sorghum bicolor* GN = SORBI_002G317600	4.45	0.12	9.13E-08	−	YjgF/YER057c/UK114 family
103	C5YW21	Malate dehydrogenase OS = *Sorghum bicolor* GN = SORBI_009G240700	5.43	0.89	2.77E-05	−	L-Lactate/malate dehydrogenase
106	C5WT90	Uncharacterized protein OS = *Sorghum bicolor* GN = SORBI_001G173300	2.48	0.28	2.43E-04	−	Reversibly glycosylated polypeptide family
113	C5XYB4	Uncharacterized protein OS = *Sorghum bicolor* GN = SORBI_004G229300	2.28	0.14	3.92E-05	−	Phosphate-induced protein 1
115	C5XQW7	Uncharacterized protein OS = *Sorghum bicolor* GN = SORBI_003G087300	2.29	0.12	8.67E-05	+	S1/P1 nuclease family
116	C5WQH5	Uncharacterized protein OS = *Sorghum bicolor* GN = SORBI_001G149500	2.79	0.48	2.33E-04	−	None predicted
117	C5Y1P6	Uncharacterized protein OS = *Sorghum bicolor* GN = SORBI_005G099500	2.44	0.15	3.59E-05	+	Nucleoside phosphatase GDA1/CD39 family
120	C5YSB1	Uncharacterized protein OS = *Sorghum bicolor* GN = SORBI_008G048400	2.55	0.21	2.60E-05	+	Alginate lyase
121	A0A1B6QAK5	Uncharacterized protein OS = *Sorghum bicolor* GN = SORBI_002G113800	2.97	0.29	3.91E-05	−	Spermidine/spermine synthases
123	C5XFH6	Fructose-bisphosphate aldolase OS = *Sorghum bicolor* GN = SORBI_003G393900	4.01	0.53	2.75E-05	−	Fructose-bisphosphate aldolase, class-I
130	C5XTG0	Uncharacterized protein OS = *Sorghum bicolor* GN = SORBI_004G166500	6.10	0.45	5.54E-07	−	N-carbamoylputrescine amidase
132	C5X9N2	Uncharacterized protein OS = *Sorghum bicolor* GN = SORBI_002G039000	4.15	0.38	4.03E-06	+	ML domain
139	C5YRS3	Purple acid phosphatase OS = *Sorghum bicolor* GN = SORBI_008G037000	3.69	0.34	5.42E-06	−	Purple acid phosphatase-like, N-terminal domain family
148	C5WT45	Uncharacterized protein OS = *Sorghum bicolor* GN = SORBI_001G168500	3.55	0.33	6.16E-06	−	Serpin family
152	C5XQ07	Uncharacterized protein OS = *Sorghum bicolor* GN = SORBI_003G072300	5.32	1.29	1.52E-04	−	Triosephosphate isomerase
165	A0A1B6PLT5	Uncharacterized protein OS = *Sorghum bicolor* GN = SORBI_006G133000	2.14	0.38	5.89E-04	+	Galactose-binding domain-like
168	C5XG88	Small ubiquitin-related modifier	6.26	1.94	6.02E-04	−	Ubiquitin-related
181	A0A1B6PJF1	Uncharacterized protein OS = *Sorghum bicolor* GN = SORBI_006G014400	2.20	0.32	8.41E-04	−	AmbAllergen

^a^Protein number assigned in ProteinPilot.

^b^Protein accession numbers obtained from the UniProt database searches against sequences of *S*. *bicolor* only.

^c^Ratio represents the average fold-change (*n* = 4) in response to sorbitol-induced osmotic stress relative to the control. A negative value indicates down-regulation.

^d^Standard deviation of the fold-changes (*n* = 4).

^e^Probability value obtained from a Student’s t-test comparing the fold changes between the sorbitol-induced osmotic stress treatments and the control (*n* = 4).

^f^Signal peptide prediction using SignalP 4.1 (http://www.cbs.dtu.dk/services/SignalP). A positive sign denotes the presence of a predicted signal peptide; a negative sign denotes the absence of a signal peptide.

^g^Family name as predicted using the InterPro (http://www.ebi.ac.uk/interpro/) and Superfamily (www.supfam.org) database.

### Analysis of sorbitol-induced gene expression

**Figure 2:**
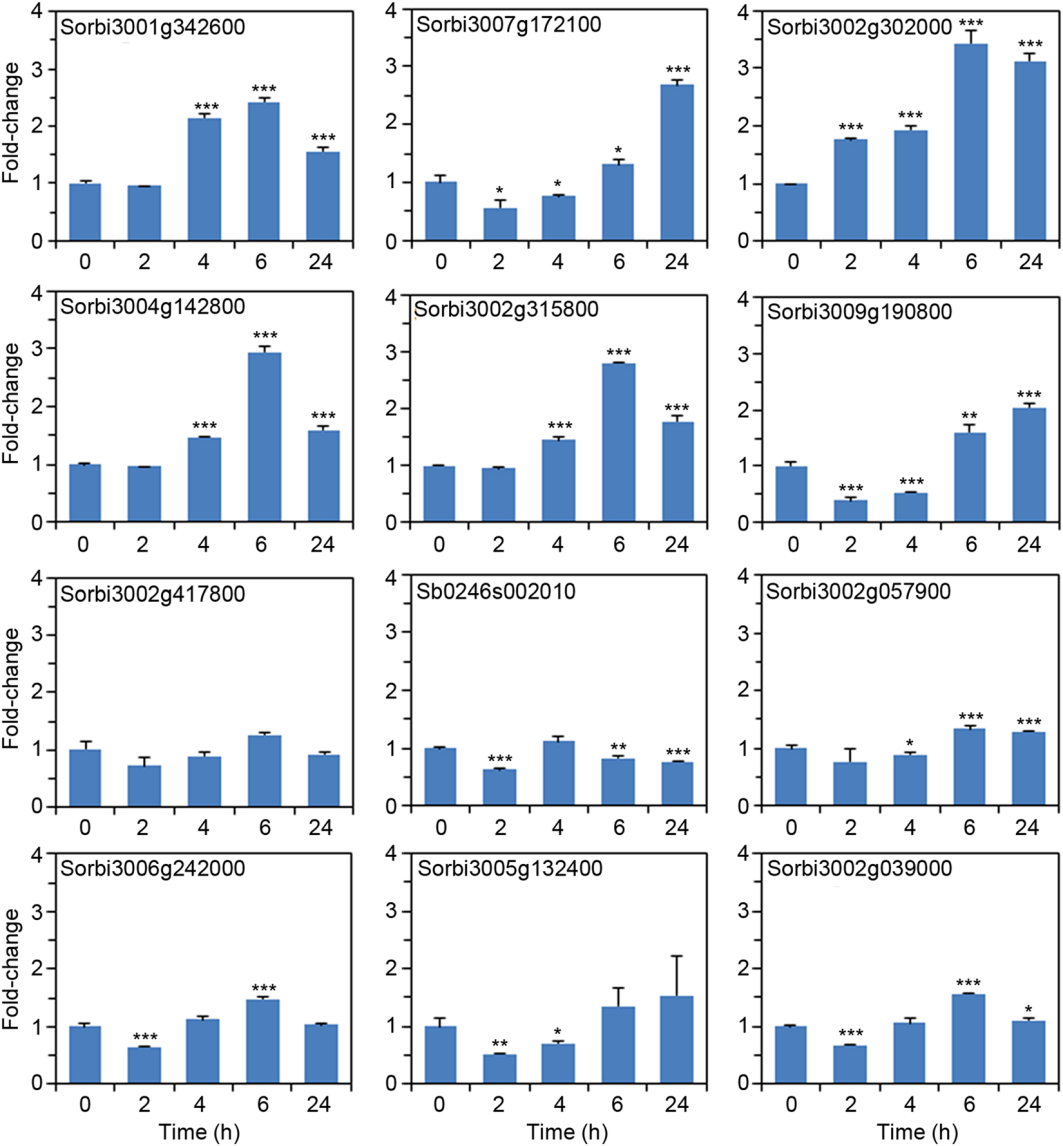
Sorbitol-induced gene expression. Sorghum cell suspension cultures were treated with sorbitol and cells harvested at the indicated time-points for qRT-PCR analysis. Error bars represent means ± S.D. (*n* = 3). One, two and three asterisks indicate statistically significant differences between control and sorbitol treatment means at each time-point, *p* ≤ 0.05, 0.01, and 0.001, respectively.

The observed increase in the amount of secreted proteins may be a result of increased expression of the genes encoding these proteins or increased translation of the corresponding mRNA. To investigate if osmotic stress transcriptionally regulated some of these candidates, we used qRT-PCR analysis on randomly selected 12 genes from the top 30 proteins of differentially expressed proteins that had been ranked in descending order of the fold-change magnitude (Supplementary Dataset - Table S3). Sorghum cell cultures were treated with sorbitol and samples for RNA extraction harvested 0, 2, 4, 6 and 24 h later. We focused on early transcriptional responses, which precede changes at the protein level analysed 48 h after sorbitol addition. With the exception of *SORBI_3002G417800*, whose expression did not respond to osmotic stress at any time-point, all the other 11 genes investigated responded significantly to sorbitol at least at one time-point (Fig. 2). However, for *Sb0246s002010* and S*ORBI_3005G132400* the significant response within the first 24 h was transcriptional repression. For the other genes, there was either an initial suppression of gene expression at the early time-points followed by activation (e.g., *SORBI_3007G172100*), or gene activation without any suppression (e.g., *SORBI_3002G302000*) (Fig. 2). Taken together, these results show that increased protein secretion into the ECM observed in this study could be driven by transcriptional regulation, post-transcriptional regulation, or regulated at both transcription and translation levels, depending on the specific proteins. Moreover, the different expression profiles across the sampled 12 genes suggest that there is complex coordination of the gene network governing the proteome response to osmotic stress.

### Analysis of drought-induced gene expression in sorghum plants

**Figure 3:**
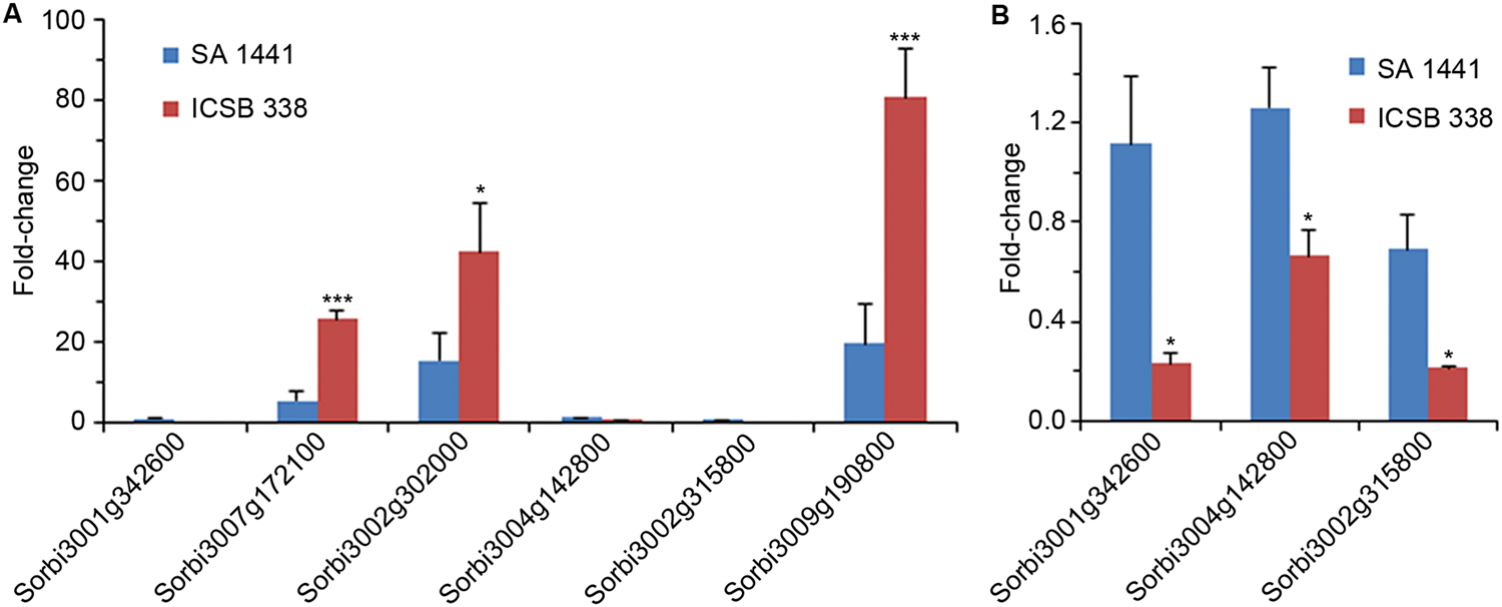
Drought stress-induced gene expression in sorghum roots. Drought-tolerant SA 1441 and drought-sensitive ICSB 338 sorghum plants were exposed to drought for 11 days and gene expression analysed by qRT-PCR. The control plants were not exposed to drought and had a gene expression value set at 1-fold. Error bars represent means ± S.D. (*n* = 5). One and three asterisks indicate statistically significant differences between the SA 1441 and ICSB 338 means, *p* ≤ 0.05 and 0.001, respectively.

Six of the 12 genes analysed by qRT-PCR were activated ≥2-fold in response to sorbitol treatment of sorghum cell suspension cultures (Fig. 2). We then investigated if activation of these 6 genes (*S0RBI_3001G342600*, *SORBI_3007G172100*, *SORBI_3002G302000*, *SORBI_3004G142800*, *SORBI_3002G315800* and *SORBI_3009G190800*) in the *in vitro* cell culture system is recapitulated in sorghum plants exposed to drought stress. We selected two sorghum varieties with contrasting drought response phenotypes; the drought-tolerant SA 1441 and “drought-sensitive” ICSB 338. After a period of growth with optimal soil water content, the plants were exposed to drought stress by withholding water for 11 days. Across all the 6 genes, there was a significant difference in drought-induced expression in root tissues of the two sorghum varieties (Fig. 3A,B). Expression of *SORBI_3007G172100*, *SORBI_3002G302000* and *SORBI_3009G190800* increased in response to drought, with up-regulation in the drought-sensitive ICSB 338 variety being significantly greater than the tolerant SA 1441 variety (Fig. 3A). Conversely, *SORBI_3001G342600*, *SORBI_3004G142800* and *SORBI_3002G315800* were significantly suppressed in the drought-sensitive ICSB 338 while remaining largely unchanged in the drought tolerant variety SA 1441 (Fig. 3B).

**Figure 4:**
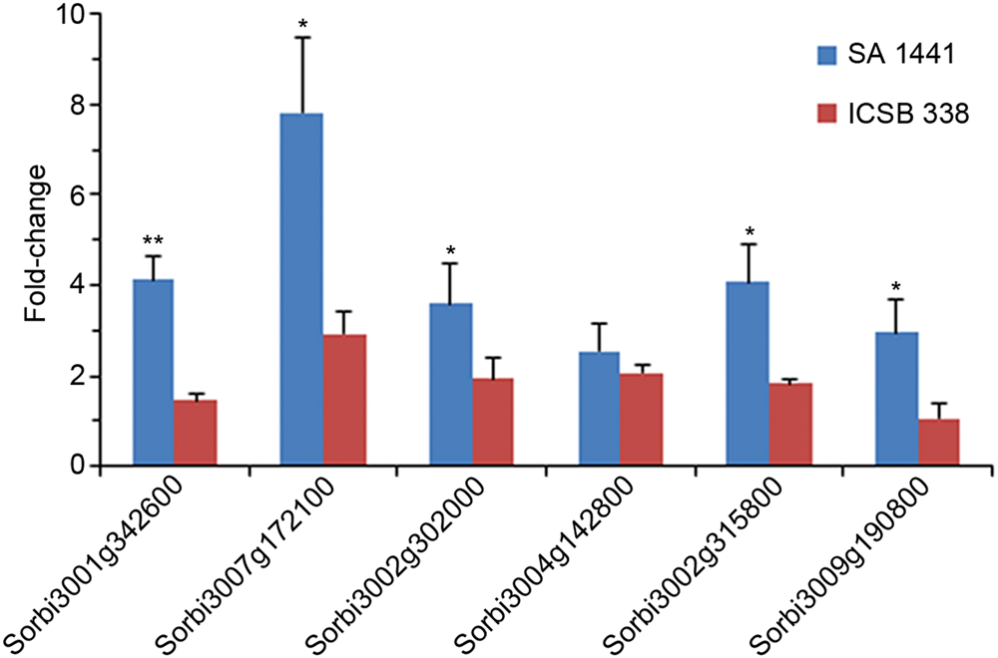
Drought stress-induced gene expression in sorghum leaves. Drought-tolerant SA 1441 and drought-sensitive ICSB 338 sorghum plants were exposed to drought for 11 days and gene expression analysed by qRT-PCR. The control plants were not exposed to drought and had a gene expression value set at 1-fold. Error bars represent means ± S.D. (*n* = 5). One and two asterisks indicate statistically significant differences between the SA 1441 and ICSB 338 means, *p* ≤ 0.05 and 0.01, respectively.

In leaf tissues, expression of all 6 genes was up-regulated in the drought-tolerant variety SA 1441 (Fig. 4). Thus, at least within this 6 gene selection, SA 1441 recruited all genes in leaf tissues responding to drought, while only half of them responded in the roots. In contrast, ICSB 338 had very marginal or no response across all genes in leaves, while the roots had a very robust upregulation of 3 genes and suppression of the other 3 genes. Collectively, these results demonstrate that candidates selected from our protein dataset are differentially expressed in sorghum lines with contrasting drought responses.

## Discussion

Drought stress triggers remarkable physiological responses and growth perturbations, which significantly diminish plant biomass and seed yield. These responses are underpinned by changes in gene expression, which are governed by poorly understood signalling processes. As sorghum is a crop that thrives under drought, it is an attractive model crop for gene discovery and studying the mechanisms driving adaptation to drought. Here we used a sorghum cell suspension culture system to obtain fractions enriched for ECM proteins. The ECM is a functional space in which secreted proteins, carbohydrates and other metabolites play a pivotal role in cell growth, cell-cell communication, and responses to changes in environmental factors. A cell culture system is scalable for production of high amounts of secretory molecules for analysis. Moreover, cell cultures are a useful *in vitro* system, which has been instrumental in key plant science discoveries, such as discovery of the roles of oxidative cross-linking of the cell wall^32^ or of ROS and nitric oxide^33,34^ in plant pathogen interactions.

We made three key observations relating to the ECM and sorghum adaptive responses to drought stress. First, there was an overall increase in protein secretion when cells were exposed to osmotic stress. Secretion of over 50% of the soluble ECM proteins identified in this study was upregulated by ≥2-fold. Similarly, an increase in protein secretion was observed in chickpea cell cultures responding to polyethylene glycol treatment^35^. Previous studies have demonstrated that increased protein secretion is essential for mounting a defensive response to pathogen attack^36,37^. Because most pathogens invade the ECM space, secretion of a cocktail of antimicrobial proteins is essential in terminating the attack. The surge in protein secretion in response to osmotic stress appears to suggest a key role for the ECM in drought adaptive responses. This might be important, particularly in switching metabolism from optimal growth to stress adaptation. Upon sensing soil water deficits, shoot growth is suppressed and resources are funnelled towards root growth in pursuit of the receding ground water. Programmed cell death may be invoked to kill off root meristems to break apical dominance^38^ as a strategy to redirect root growth away from water-depleted zones towards available water gradients. The changes in protein expression observed here constitute part of the gene network underpinning these physiological and morphological changes. Proteins are part of the molecular cargo exported into the plant ECM to build the cell wall infrastructure, decorate the external face of the plasma membrane with receptor complexes, and regulate cell division and differentiation^30,39^. The heightened protein secretion triggered by osmotic stress could play a crucial role in mediating the changes in growth and cellular physiology associated with drought.

The second key finding relates to identification of specific differentially expressed ECM proteins. These fell into four broad functional categories, namely glycosyl-hydrolases/glycosidases, cell wall modifying enzymes, proteases, and redox proteins. Glycosyl-hydrolases/glycosidases are known carbohydrate metabolising enzymes and have diverse substrate specificity^40,41^. In this study, we identified 18 hydrolases from different families, indicating the wide spectrum of substrate specificity and mechanisms of action. Although the precise role of these enzymes in osmotic stress response is not clear, carbohydrates are important biomolecules, which have structural^42^ and signalling^40^ functions. Interestingly, none of these glycosyl hydrolases/glucosidases identified in the present secretome study were reported in a sorghum drought study, which focused on the leaf proteome^28^. However, glycosyl-hydrolases/glycosidases have also been identified in secretome studies of Arabidopsis responding to both pathogen attack^43^ and nutritional phosphate deficiency^44^. A computational functional annotation study attempted to assign putative functions to the 238 uncharacterised sorghum glycoside hydrolases, with stress response functions being ascribed to these enzymes^45^.

There were 5 cell wall modifying proteins that responded to osmotic stress, which included putative expansin-like and fascilin-like protein families (Table 1). Expansins are known extracellular proteins involved in remodelling cell walls by facilitating cell wall relaxation and extension^46^; while fascilin domain containing proteins may be involved in cell adhesion processes^47^. Expansins have been identified in rice secretome studies exposed to rice blast fungus and elicitor^48^, while a fascilin-like arabinogalactan protein was identified in Arabidopsis secretome following pathogen infection^43^. Our study indicates that the role for these proteins span several types of plant stress.

Of particular note was the increased secretion of proteases and redox proteins. The identified proteases are putative members of the peptidase, serine carboxypeptidase, aspartic peptidase, gamma-glutamyl-transpeptidase and peptidase subtilisin-related protein families. Proteolytic cleavage of proteins and peptides could be useful in regulating enzyme activity^49^ and post-translational activation of peptide signals via cleavage of inhibitory domains of pro-peptides^50,51^. Deployment of these signal regulatory proteins could play critical roles during stress adaptation. Proteolysis could also function in the control of protein turnover, which becomes critical during stress response^52^. These enzymes have also been identified in previous secretome studies^31,44^. Several redox proteins, including peroxidases and thioredoxin had increased secretion after imposition of osmotic stress. Peroxidases are important in cell wall lignification^53^, but are also part of a large protein network that controls the homeostasis of ROS. At low concentration, ROS serve a signalling role^54,55^, but function in cell death activation at high concentration^55,56^. Thioredoxin is a molecular switch used for regulating enzyme activity via reducing disulphide bridges linking cycteine residues^57,58^. Overall, our results indicate that ECM protein networks could play very wide-ranging functions in drought stress adaptive responses.

The third key observation we made was that genes encoding selected candidate proteins are differentially expressed between drought-tolerant and “drought-sensitive” sorghum varieties exposed to drought. We found that selected genes are transcriptionally regulated by sorbitol-induced osmotic stress in the *in vitro* cell suspension culture system. Analysis of these genes in sensitive versus drought-tolerant sorghum varieties exposed to drought revealed significant differences in expression profiles. Drought activation of gene expression in the sensitive sorghum line was limited to 3 genes mainly in root tissues, with very modest changes in leaves (Figs 3 and 4). Although activation of the same 3 genes in roots of the drought-tolerant SA 1441 was lower than in ICSB 338, the latter also activated all six genes in leaves (Fig. 4). This may indicate that successful drought tolerance requires adaptive gene expression in both subterranean and aerial plant organs. Future genetic experiments could provide functional data of single or multiple genes in adaptive responses to drought. Collectively, these expression profiles indicate two things. First, that the ECM could provide targets for use in enhancing drought tolerance in crops. Because the response of the sorghum varieties to drought differs from each other, then genes/proteins differing in their response to drought between the two varieties could be potential key regulators of drought adaptation. Second, that datasets of differentially expressed ECM proteins under osmotic stress may provide biomarkers that could be used in breeding programmes to rapidly identify drought-tolerant and sensitive varieties.

In conclusion, the ECM is replete with proteins involved in cell growth control, cell communication and cell signalling during responses to environmental stress. A wide range of plant species and experimental systems has been used to study the ECM proteins, including sorghum. This study extends the number of proteins identified in the sorghum ECM from 14 proteins^29^ to 179 proteins (Supplementary Dataset - Table S2). A large proportion of these (∼72%) possess a predicted signal peptide (Supplementary Dataset - Table S2), which targets them to the secretory pathway^59^, while the remainder do not possess a signal peptide. This raises the concern of whether the apparent increase in secretion of some of these proteins actually arises from sorbitol-induced cell death and release of intracellular proteins. However, we discount this possibility on the basis of three observations. First, all the proteins identified with increased abundance after sorbitol treatment were also identified in the stress-free control cell cultures. Their secretion in exponentially growing viable control cultures makes cell death an unlikely cause. Secondly, if cell death was responsible, we would have expected to identify many abundant cytosolic house-keeping proteins appearing in sorbitol samples only and absent from control samples. This was not the case. Finally, sorbitol at the concentrations used and time-scale of treatment causes cells to lose water and shrink, with no reduction in cell viability (data not shown). Proteins without a signal peptide identified in our ECM fractions add to the growing number of animal and plant proteins, which are secreted into the ECM via alternative mechanisms not requiring the signal peptide^60,61^. For example, a leaderless CaRRP1 protein has been confirmed to be a *bona fide* ECM protein using a YFP-tagged recombinant version of the protein^35^. Increased secretion of both signal peptide-containing and leaderless proteins is a strong indication that the ECM protein network is part of the molecular machinery deployed when sorghum encounters deficits in soil water content. Importantly, differential expression of some of the target proteins between drought-tolerant and drought-sensitive sorghum varieties implicates the candidates in mediating drought tolerance, though genetic experiments will be required to definitively confirm this.

## Methods

### Plant material and growth conditions

Seeds of white sorghum previously used for the generation of cell suspension cultures^62^ were obtained from Professor Bongani Ndimba (Agricultural Research Council (ARC), South Africa). In this study, white sorghum callus and cell suspension cultures were initiated and maintained on Murashige and Skoog Minimal Organics medium under dark conditions as described previously^62^. The cell cultures were sub-cultured every two weeks and used for sorbitol-induced osmotic stress treatments 8 days post sub-culture. Drought-tolerant (SA 1441) and drought-sensitive (ICSB 338) sorghum varieties were obtained from the ARC-Grain Crop Institute, Potchefstroom, South Africa. Sorghum seeds were sown in potted soil and grown at 25–30 °C under a 16 h-photoperiod. Plants were grown in square pots with a volume of 216 cm^3^ filled with Levington F2 + Sand compost (ICL Ltd., Ipswich, UK). All plants were well watered until they reached the V3 stage (3 fully expanded leaves with the fourth one emerging) before imposing drought stress by cessation of watering.

### Osmotic and drought stress treatments

Eight days after subculturing, sorghum cell suspension cultures were exposed to osmotic stress by treating with 400 mM sorbitol. Control cell cultures were spiked with an equivalent volume of sterile distilled water for the same duration. A time-course sorbitol treatment experiment of sorghum cell suspension cultures was carried over a 72 h period, and expression analysis of drought marker genes *ERD1* (early responsive to dehydration 1) and *DREB2A* was analysed at 0, 24, 48 and 72 h in order to establish the most appropriate time for proteome analysis. For protein analysis, 4 biological replicates of 30 mL each were treated with sorbitol and harvested 48 h later. For RNA analysis, 3 biological replicates of 10 mL each were treated and harvested 0, 2, 4, 6, and 24 h later. For drought stress treatments, well-watered plants at the V3 stage were divided into two groups; control and drought stressed plants. The control plants were watered throughout the experiment as necessary, while water was withheld for 11 days from the drought-stressed plants. Five biological replicates were generated for each group. For leaf samples, each biological replicate was a pool of 3 leaves, each coming from an independent plant. For root samples, a biological replicate consisted of roots pooled from 2 plants. The leaf or root material was snap-frozen in liquid nitrogen and stored at −80 °C for use in RNA extraction.

### RNA extraction and analysis

Total RNA was extracted from cell cultures, root and leaf samples using RNeasy Plant Kits (Qiagen, Manchester, UK) according to the manufacturer’s instructions. First strand complementary DNA (cDNA) synthesis was performed using 1.5 µg total RNA template and oligo-(dT)_15_ using the GoScript^TM^ Reverse Transcription System (Promega, Southampton, UK) according to the manufacturer’s instructions. Quantitative real-time PCR (qRT-PCR) was performed on the Rotar-Gene 3000 (Corbett Research, Sydney, Australia) using the SensiFAST™ SYBR® No-ROX kit (Bioline, London, UK). The reaction consisted of 10 µl SensiFAST reagent, 0.4 µM each of the forward and reverse primers, and 5 µl of 8-fold dilution cDNA in a final volume of 20 µl. The thermal cycling conditions were as follows: denaturation at 95 °C for 3 min followed by 40 cycles of 95 °C for 10 sec, annealing at 56 °C for 15 sec and extension at 72 °C for 25 sec. All reactions were carried out on 3 technical replicates for each of the biological replicate. Data analysis was carried out using the REST2009 version 2.0.13 software (Qiagen) using *Sb03g038910* as a constitutive reference control gene, whose expression does not alter in response to drought stress^27^. The primer sequences of all genes used are listed in supplementary Table S1.

### Protein sample preparation and iTRAQ Labelling

Control and sorbitol-treated cell cultures were filtered through 2 layers of Miracloth to separate the cells from the growth medium. Secreted proteins were isolated from the growth medium by acetone precipitation as described previously^63^ and solubilised in a solution containing 9 M urea, 2 M thiourea and 4% (w/v) CHAPS. There were 4 biological replicates of controls and the same for sorbitol treatments. Labelling of protein samples with iTRAQ tags was performed as described previously^63^ with minor modifications. Briefly, for each sample, 50 μg of protein were reduced with tris(2-carboxyethylphosphine) (TCEP) and alkylated with methyl-methane-thiol-sulfonate (MMTS). Thereafter, protein samples were digested at 37 °C for ~16 h using a 1:10 (w/w) trypsin to protein sample ratio, vacuum-dried, re-suspended in triethylammonium bicarbonate buffer (pH 8.5), and labelled with an 8-plex iTRAQ reagent kit (Applied Biosystems Sciex, Foster City, USA) according to the manufacturer’s instructions.

Peptides of the 4 control replicates were labelled with 113, 114, 115, and 116 iTRAQ tags, while sorbitol-treated samples were labelled with 117, 118, 119, and 121 tags. All eight samples were pooled to make one composite sample, which was then vacuum-dried and re-suspended in 3.8 mL of buffer A (10 mM K_2_HPO_4_/25% acetonitrile, pH 3.0). Thereafter, the sample was separated into 50 fractions on a PolySULFOETHYL A strong cation exchange column (Poly LC Inc. 200 × 2.1 mm, 5 μm) at 300 nL/min on an Ettan LC (GE Healthcare, Pittsburgh, USA) HPLC system. Peptide separation was performed using a biphasic gradient of: 0–150 mM KCl over 11.25 column volumes and 150–500 mM KCl in buffer A over 3.25 column volumes. A total of 50 fractions were collected over the gradient, and reduced to 30 by pooling those with low peptide concentration. The 30 fractions were dried down and re-suspended in 90 μL of 2% acetonitrile/0.1% formic acid. Aliquots of 20 μL from each fraction were analysed by LC-MS/MS using a QStar Pulsar *i* mass spectrometer (MDS-Sciex/Applied Biosystems).

### Mass spectra data analysis

Mass spectra data were analysed as described previously^63^, with minor modifications. Briefly, ProteinPilot software 4.5 (Beta) Revision 1656 Paragon algorithm build 1654 (ABSciex) was used for data analysis against the UniProt database sequences for *S*. *bicolor* (downloaded in October 2013, 58756 entries) plus 162 known contaminants from proteomic experiments. A minimum score threshold of 2.0 (99% confidence) was set for protein identification and all proteins identified on the basis of a single peptide were filtered out of the dataset, resulting in a total of 179 unique proteins.

For quantitative analysis of the differentially expressed proteins, the abundance of each protein in all samples was calculated as a ratio to the 113-tagged control sample. Averages of the ratios for each protein across the four replicates were calculated. The fold-change in protein expression was denoted by the ratio of control to sorbitol-treated samples. For the down-regulated proteins, the osmotic stressed average was the numerator and the control was the denominator, with a negative sign denoting down-regulation. A probability value for the comparison of the control average to sorbitol average was obtained from the Student’s *t*-test at 95% confidence.

### Bioinformatic analysis

The presence of an N-terminal signal peptide on all identified proteins was predicted using SignalP 4.1^64^. The InterPro^65^ and Superfamily^66^ databases were used for protein sequence analysis to identify protein functional domains used for assignments to relevant protein families.

### Data availability statement

The datasets generated and/or analysed during the current study are available from the corresponding author on request.

## Electronic supplementary material

Table S1

Dataset 1

## References

[1] Harris MJ, Outlaw WH, Mertens R, Weiler EW (1988). Water-stress-induced changes in the abscisic acid content of guard cells and other cells of *Vicia faba* L. leaves as determined by enzyme-amplified immunoassay. P. Natl. Acad. Sci. USA.

[2] Jones RJ, Mansfield TA (1970). Suppression of stomatal opening in leaves treated with abscisic acid. J. Exp. Bot..

[3] Shinozaki K, Yamaguchi-Shinozaki K (1997). Gene expression and signal transduction in water-stress response. Plant Physiol..

[4] Park SY (2009). Abscisic acid inhibits type 2C protein phosphatases via the PYR/PYL family of START proteins. Science.

[5] Ma Y (2009). Regulators of PP2C phosphatase activity function as abscisic acid sensors. Science.

[6] Melcher K (2009). A gate-latch-lock mechanism for hormone signalling by abscisic acid receptors. Nature.

[7] Miyazono K (2009). Structural basis of abscisic acid signalling. Nature.

[8] Nishimura, N. *et al*. Structural mechanism of abscisic acid binding and signaling by dimeric PYR1. *Science* **326** (2009). 10.1126/science.1181829 PMC2835493 19933100

[9] Santiago J (2009). The abscisic acid receptor PYR1 in complex with abscisic acid. Nature.

[10] Yin P (2009). Structural insights into the mechanism of abscisic acid signaling by PYL proteins. Nat. Struct. Mol. Biol..

[11] Zhang X (2012). Complex structures of the abscisic acid receptor PYL3/RCAR13 reveal a unique regulatory mechanism. Structure.

[12] Fujii H (2009). *In vitro* reconstitution of an abscisic acid signalling pathway. Nature.

[13] Soon FF (2012). Molecular mimicry regulates ABA signaling by SnRK2 kinases and PP2C phosphatases. Science.

[14] Ng LM (2011). Structural basis for basal activity and autoactivation of abscisic acid (ABA) signaling SnRK2 kinases. P. Natl. Acad. Sci. USA.

[15] Okamoto M (2013). Activation of dimeric ABA receptors elicits guard cell closure, ABA-regulated gene expression, and drought tolerance. P. Natl. Acad. Sci. USA.

[16] Busk PK, Pages M (1998). Regulation of abscisic acid-induced transcription. Plant Mol. Biol..

[17] Abe H (1997). Role of arabidopsis MYC and MYB homologs in drought- and abscisic acid-regulated gene expression. Plant Cell.

[18] Fujita Y (2005). AREB1 is a transcription activator of novel ABRE-dependent ABA signaling that enhances drought stress tolerance in Arabidopsis. Plant Cell.

[19] Abe H (2003). Arabidopsis AtMYC2 (bHLH) and AtMYB2 (MYB) function as transcriptional activators in abscisic acid signaling. Plant Cell.

[20] Yamaguchi-Shinozaki K, Shinozaki K (1994). A novel cis-acting element in an Arabidopsis gene is involved in responsiveness to drought, low-temperature, or high-salt stress. Plant Cell.

[21] Liu Q (1998). Two transcription factors, DREB1 and DREB2, with an EREBP/AP2 DNA binding domain separate two cellular signal transduction pathways in drought- and low-temperature-responsive gene expression, respectively, in Arabidopsis. Plant Cell.

[22] Sakuma Y (2006). Dual function of an Arabidopsis transcription factor DREB2A in water-stress-responsive and heat-stress-responsive gene expression. P. Natl. Acad. Sci. USA.

[23] Holmberg N, Bulow L (1998). Improving stress tolerance in plants by gene transfer. Trends Plant Sci..

[24] Rosenow DT, Quisenberry JE, Wendt CW, Clark LE (1983). Drought Tolerant Sorghum and Cotton Germplasm. Agr. Water Manage..

[25] Ngara R, Ndimba BK (2014). Model plant systems in salinity and drought stress proteomics studies: a perspective on Arabidopsis and Sorghum. Plant Biology.

[26] Paterson AH (2009). The *Sorghum bicolor* genome and the diversification of grasses. Nature.

[27] Johnson SM (2014). Transcriptomic analysis of *Sorghum bicolor* responding to combined heat and drought stress. BMC Genomics.

[28] Jedmowski C (2014). Comparative Analysis of *Sorghum bicolor* Proteome in Response to Drought Stress and following Recovery. Int. J. Proteomics.

[29] Ngara R, Ndimba BK (2011). Mapping and characterisation of the sorghum cell suspension culture secretome. Afr. J. Biotechnol..

[30] Chivasa S, Slabas AR (2012). Plant extracellular ATP signalling: new insight from proteomics. Molecular Biosyst..

[31] Ndimba BK, Chivasa S, Simon WJ, Slabas AR (2005). Identification of Arabidopsis salt and osmotic stress responsive proteins using two-dimensional difference gel electrophoresis and mass spectrometry. Proteomics.

[32] Brisson LF, Tenhaken R, Lamb C (1994). Function of oxidative cross-linking of cell wall structural proteins in plant disease resistance. Plant Cell.

[33] Delledonne M, Xia Y, Dixon RA, Lamb C (1998). Nitric oxide functions as a signal in plant disease resistance. Nature.

[34] Delledonne M, Zeier J, Marocco A, Lamb C (2001). Signal interactions between nitric oxide and reactive oxygen intermediates in the plant hypersensitive disease resistance response. P. Natl. Acad. Sci. USA.

[35] Gupta S (2015). Secretome analysis of chickpea reveals dynamic extracellular remodeling and identifies a Bet v1-like protein, CaRRP1 that participates in stress response. Sci. Rep..

[36] Kwon C, Bednarek P, Schulze-Lefert P (2008). Secretory pathways in plant immune responses. Plant Physiol..

[37] Kwon C (2008). Co-option of a default secretory pathway for plant immune responses. Nature.

[38] Duan Y (2010). An endoplasmic reticulum response pathway mediates programmed cell death of root tip induced by water stress in Arabidopsis. New Phytol..

[39] Wang D, Dong X (2011). A highway for war and peace: the secretory pathway in plant-microbe interactions. Mol. Plant..

[40] Davies G, Henrissat B (1995). Structures and mechanisms of glycosyl hydrolases. Structure.

[41] Henrissat B, Davies G (1997). Structural and sequence-based classification of glycoside hydrolases. Curr. Opin. Struc. Biol..

[42] Garrett, R., Grisham, C. M. & Sabat, M. *Biochemistry*. 4th. edn, (Brooks/Cole Pub Co, 2010).

[43] Kaffarnik FA, Jones AM, Rathjen JP, Peck SC (2009). Effector proteins of the bacterial pathogen *Pseudomonas syringae* alter the extracellular proteome of the host plant, *Arabidopsis thaliana*. Mol. Cell. Proteomics.

[44] Tran HT, Plaxton WC (2008). Proteomic analysis of alterations in the secretome of *Arabidopsis thaliana* suspension cells subjected to nutritional phosphate deficiency. Proteomics.

[45] Sekhwal MK, Sharma V, Sarin R (2013). Annotation of glycoside hydrolases in *Sorghum bicolor* using proteins interaction approach. J. Proteome Sci. Comput. Biol..

[46] Cosgrove DJ (2000). New genes and new biological roles for expansins. Curr. Opin. Plant Biol..

[47] Johnson KL, Jones BJ, Bacic A, Schultz CJ (2003). The fasciclin-like arabinogalactan proteins of Arabidopsis. A multigene family of putative cell adhesion molecules. Plant Physiol..

[48] Kim ST (2009). Secretome analysis of differentially induced proteins in rice suspension-cultured cells triggered by rice blast fungus and elicitor. Proteomics.

[49] Vierstra RD (1996). Proteolysis in plants: mechanisms and functions. Plant Mol. Biol..

[50] McGurl B, Pearce G, Orozco-Cardenas M, Ryan CA (1992). Structure, expression, and antisense inhibition of the systemin precursor gene. Science.

[51] Lindsey K (2001). Plant peptide hormones: The long and the short of it. Curr. Biol. CB.

[52] Liu H (2008). A rice serine carboxypeptidase-like gene OsBISCPL1 is involved in regulation of defense responses against biotic and oxidative stress. Gene.

[53] Hiraga S, Sasaki K, Ito H, Ohashi Y, Matsui H (2001). A large family of class III plant peroxidases. Plant Cell Physiol..

[54] Hancock JT, Desikan R, Neill SJ (2001). Role of reactive oxygen species in cell signalling pathways. Biochemical Soc. Trans..

[55] Desikan R, Reynolds A, Hancock JT, Neill SJ (1998). Harpin and hydrogen peroxide both initiate programmed cell death but have differential effects on defence gene expression in Arabidopsis suspension cultures. Biochemical J..

[56] Levine A, Pennell RI, Alvarez ME, Palmer R, Lamb C (1996). Calcium-mediated apoptosis in a plant hypersensitive disease resistance response. Curr. Biol..

[57] Arner ES, Holmgren A (2000). Physiological functions of thioredoxin and thioredoxin reductase. Eur. J. Biochem..

[58] Meyer Y (2008). Glutaredoxins and thioredoxins in plants. Biochim. Biophys. Acta.

[59] Nielsen H, Engelbrecht J, Brunak S, von Heijne G (1997). Identification of prokaryotic and eukaryotic signal peptides and prediction of their cleavage sites. Protein Eng..

[60] Krause C, Richter S, Knoll C, Jurgens G (2013). Plant secretome - from cellular process to biological activity. Biochim. Biophys. Acta.

[61] Ding Y, Robinson DG, Jiang L (2014). Unconventional protein secretion (UPS) pathways in plants. Curr. Opin. Cell Biol..

[62] Ngara R, Rees J, Ndimba BK (2008). Establishment of sorghum cell suspension culture system for proteomics studies. Afr. J. Biotechnol..

[63] Smith SJ, Kroon JT, Simon WJ, Slabas AR, Chivasa S (2015). A Novel Function for Arabidopsis CYCLASE1 in Programmed Cell Death Revealed by Isobaric Tags for Relative and Absolute Quantitation (iTRAQ) Analysis of Extracellular Matrix Proteins. Mol. Cell. Proteomics.

[64] Petersen TN, Brunak S, von Heijne G, Nielsen H (2011). SignalP 4.0: discriminating signal peptides from transmembrane regions. Nature Methods.

[65] Mulder, N. J. *et al*. New developments in the InterPro database. *Nucleic Acids Res*. **35** (2007). 10.1093/nar/gkl841 PMC1899100 17202162

[66] Wilson, D. *et al*. SUPERFAMILY–sophisticated comparative genomics, data mining, visualization and phylogeny. *Nucleic Acids Res*. **37** (2009). 10.1093/nar/gkn762 PMC2686452 19036790

