# Supplementary material for: Identifying differentially expressed proteins in sorghum cell cultures exposed to osmotic stress
Source: Sci Rep. 2018 Jun 6;8:8671. doi: 10.1038/s41598-018-27003-1 (PMC5989219; doi:10.1038/s41598-018-27003-1)
Supplement: Supplementary file 1 — Table S1 [file 41598_2018_27003_MOESM1_ESM.pdf]

Title: Identifying differentially expressed proteins in sorghum cell cultures exposed to osmotic stress

Authors: Rudo Ngara, Elelwani Ramulifho, Mahsa Movahedi, Nemera G. Shargie, Adrian P. Brown, & Stephen Chivasa

Supplementary Table S1. Primer sequences of sorghum target genes used in quantitative real time PCR analysis

Target gene[a]	Forward primer	Reverse primer
SORBI_3004G162400	5′-AGGGACGACAGAGCATAGG-3′	5′-TGGCCAGCATCTGAGTCTTC-3′
SORBI_3006G065100	5′-GTGCATTGCGTCGACAACTC-3′	5′-CCTCCTGGCTAGGCTCATTG-3′
SORBI_3009G101400	5′-TCCTGAGGGGTCAAAACGAC-3′	5′-TTGTCAGGCCCTCCTTTTCC-3′
SORBI_3003G058200	5′-TTGTCAGGAAGAAGAGGATGAGGAG-3′	5′-TTTCTTGGAACCCTTGGCCG-3′
SORBI_3001G342600	5′-AAAGGCCCAGCTGATGATCC-3′	5′-CGTCATGCAGCTCATGCTTC-3′
Sb0246s002010	5′-ATAGCTCTCTGGCTCCTGACG-3′	5′-CGCTTCTCGCTGTCGATGG-3′
SORBI_3002G057900	5′-ATGAGGCCACCAACGCAATC-3′	5′-ACGCGATCACGACTCCCTC-3′
SORBI_3004G142800	5′-ACATACATCGGAGGCGTGTC-3′	5′-TATGGCTTGTCCTTCAGGCG-3′
SORBI_3002G315800	5′-CTTTTGGTGCTGTGGAGTGC-3′	5′-CCATCGCCGCACATAAAACC-3′
SORBI_3009G190800	5′-TGGTCCCACGGGATAAATTCG-3′	5′-CTTGAGAAGCTGAGGAGCGG-3′
SORBI_3005G132400	5′-GACAAGCCTATCGGCAAACC-3′	5′-TTCTCGTCAGCAGCAGAACC-3′
SORBI_3002G039000	5′-TCATCAGCAGACGTTACCAGG-3′	5′-CAATGCTGAACCCGAACGAG-3′
SORBI_3002G417800	5′-GGTCCCCTGTCTGGAAATCG-3′	5′-GATCCATCAAGGCCTACCGC-3′
SORBI_3007G172100	5′-GGATCAACGCGATCAAGACG-3′	5′-GCTTGGCGATGTACTGGAAC-3′
SORBI_3002G302000	5′-TCTCCACTCGCATCAAGCTC-3′	5′-TCGATCTCGTCGTGGAAACC-3′
SORBI_3006G242000	5′-TCACCAACGAAGAGTACCGC-3′	5′-CAGGTCCTCGTTATCGTCGG-3′
Sb03g038910*	5′-TCCTGAAGCATCTTTCCCTCC-3′	5′- ACAGCCTGATTAGTTGGGGG-3′

[a]Target gene locus identity obtained from the Uniprot database.
*Reference control gene, whose expression does not change in response to drought stress[27].
